# Potentially Probiotic *Lactobacillus* Strains Derived from Food Intensify Crystallization Caused by *Proteus mirabilis* in Urine

**DOI:** 10.1007/s12602-020-09689-w

**Published:** 2020-08-04

**Authors:** Agnieszka Torzewska, Paulina Wiewiura, Dominika Brodecka, Dominika Szczerbiec, Antoni Różalski

**Affiliations:** Department of Biology of Bacteria, Faculty of Biology and Environmental Protection, University of Lodz, Banacha 12/16, 90-237 Lodz, Poland

**Keywords:** *Proteus mirabilis*, Urolithiasis, Crystallization, *Lactobacillus*, Probiotics, Interspecies interaction

## Abstract

*Proteus mirabilis* is a common cause of infectious urolithiasis. The first stage in the formation of urinary stones is the crystallization of mineral salts in the urine induced by urease activity of this microorganism. *Lactobacillus* spp*.* are an important component of the human microbiota and in large quantities occur in foods. Regardless of their origin, those with probiotic properties are proposed as an alternative to antibiotic therapy in the treatment of urinary tract infections. The aim of the study was to check the effect of selected *Lactobacillus plantarum* and *Lactobacillus brevis* strains on crystallization caused by *P. mirabilis* in an in vitro experiment. It has been confirmed that selected *Lactobacillus* strains have antibacterial properties and colonize the urinary tract epithelium. During 24-h incubation of bacterial cultures, containing *P. mirabilis* and individual *Lactobacillus* strains, in synthetic urine, bacterial viability (CFU/mL), pH, and crystallization were determined. Crystallization was assessed quantitatively and qualitatively using AAS and XRD techniques as well as phase-contrast microscopy. It has been shown that in the presence of selected *Lactobacillus* strains, the culture pH increases faster, especially after 8 h of incubation, compared with the pure *P. mirabilis* culture. An increase in pH reduces the viability of *P. mirabilis*; however, in the presence of some lactobacilli, the uropathogen grows more intensively. The presence of *Lactobacillus* also affected crystallization by increasing its intensity, and the resulting crystals were larger in size. Tested *L. plantarum* and *L. brevis* strains could therefore accelerate the formation of urinary stones and development of infection.

## Introduction

*Proteus* spp. belong to the family *Morganellaceae* and occur both in the natural environment and as a part of the microflora of humans and animals [1, 36]. These bacilli which are found in water, soil, and manure play an important role in decomposing the organic matter of animal origin. However, first of all, they are known for their pathogenicity. *Proteus* spp., mainly *Proteus mirabilis*, play a particularly important role in urinary tract infections (UTI), primarily in long-term catheterized patients or patients with functional and structural abnormalities of the urinary tract. *Proteus* infections are known to be frequently persistent and difficult to treat and can lead to several complications such as bacteriuria, acute or chronic pyelonephritis, and urinary stone formation [5]. Urolithiasis resulting from urinary tract infections, called also infectious urinary stones, is distinguished by several features from metabolic urolithiasis. These stones are formed as a result of infection caused by urease-producing bacteria and made off struvite (magnesium ammonium phosphate) and apatite (calcium phosphate, mainly carbonate apatite) [10, 15]. They are not very common compared to other types of urinary stones (about 10–15% of all urinary calculi); however, they pose a serious health problem because of difficulties in treating and preventing relapse [30]. Among urease-producing uropathogens, *P. mirabilis* is most frequently isolated from such stones [32, 42]. The process of kidney stones formation consists of three main stages: supersaturation of urine salts, formation of crystals, and their retention in the urinary tract [8]. Each stage in the process of infectious stones formation is a result of the presence of bacteria in the urinary tract. Supersaturation and crystallization of mineral salts are connected with the urease activity. Urease hydrolyzes urea to ammonia and carbamate, which is unstable and forms carbonic acid and ammonia. An increase in urine pH and the reactions occurring as a result of urea hydrolysis cause an increase in the concentration of ions: NH^4+^, PO_4_^3−^, and CO_3_^2−^, which in the presence of calcium and magnesium ions in the urine leads to precipitation of phosphate salt and promotes crystallization of struvite (MgNH_4_PO_4_ × 6 H_2_O) and carbonate apatite (Ca_10_(PO_4_)_6_ × CO_3_) [34, 35]. Further stages are the growth of crystals and retention of their aggregates in the urinary tract in which bacterial extracellular polysaccharides (lipopolysaccharide; LPS, capsular polysaccharide; CPS) and various host factors released from the inflammatory response are involved [30, 39]. Treating patients with infectious urolithiasis is complex and includes stone removal (percutaneous nephrolithotomy “PCNL”; shockwave lithotripsy “SWL”), pathogen elimination, and relapse prevention [15]. Antibiotic therapy is carried out for up to 3 months to eliminate bacteria from the urinary tract. The antibiotic is chosen according to the sensitivity of bacteria, but in the case of this type of infection, it is recommended to administer of fluoroquinolones [10, 15, 20]. To prevent recurrence, the treatment is supplemented by acidifying the urine, administering bacterial urease inhibitors (e.g., acetohydroxamic acid), and an appropriate diet [10, 38]. Infectious urolithiasis is more common in people who are more likely to get urinary tract infections, due to urinary tract abnormalities or predisposition to this kind of infections. It is more common among women than men because of their higher susceptibility. On the other hand, among children, boys have struvite stones more often than girls [35]. It seems that the most important procedure in the treatment and prevention of infectious urolithiasis is combating both infection and crystallization. In the fight against infection, other alternative antibacterial agents are used in addition to antibiotics. Recently, much attention has been paid to the use of probiotic concepts or only their products in the treatment of patients with chronic urinary tract infections [16, 17]. The efficacy of probiotics against various uropathogens such as *Proteus* spp*.*, *K. pneumoniae*, *E. coli*, or *E. faecalis* was confirmed by in vitro and in vivo studies [14, 17, 23, 41]. Antagonistic effect on uropathogens has been proven for many types of probiotic microorganisms among others *Lactobacillus*, *Bifidobacterium*, *Saccharomyces*, *Pediococcus*, and *Bacillus* [2, 18, 23, 25]. Of the genus *Lactobacillus*, many species isolated from various sources such as genitourinary tract, faces, and fermented food exhibit antagonistic activity against uropathogens, for example, *L. gasseri*, *L. crispatus*, *L. rhamnosus*, *L. plantarum*, *L. brevis*, and *L. acidophilus* [23, 26, 33, 41]. Their use in women with bacterial vaginosis and recurrent UTI also brings positive effects [7]. The human body is intentionally colonized by administering selected probiotic organisms but also by consuming food containing these bacteria. The mechanism preventing the development of urinary tract infections by *Lactobacillus* spp*.* consists mainly in the production of substances with antibacterial activity, inhibiting adhesion and immunomodulatory activity [29, 41]. Among the antibacterial substances produced by *Lactobacillus* spp., besides lactic acid, should be mentioned: biosurfactants, hydrogen peroxide, bacteriocins, and other organic acids [41].

The effect of *Lactobacillus* on the viability or virulence factors of uropathogens has been established so far, but it has not been investigated how these microorganisms affect one of the complications of urinary tract infections, which is the development of infectious urolithiasis. The purpose of this work was to determine the effect of *Lactobacillus brevis* and *Lactobacillus plantarum* on the crystallization caused by *Proteus mirabilis*, which initiates the formation of urinary stones.

## Material and Methods

### Bacterial Strains

*Lactobacillus* strains were isolated from fermented cucumbers, which are the most popular fermented foods consumed in Poland. Juice of fermented cucumbers was seeded on MRS agar (De Man, Rogosa and Sharpe agar, BTL, Warsaw, Poland) and incubated in aerobic condition for 24 h at 37 °C. Bacteria were isolated and identified from selected colonies. Isolates which were Gram-positive and catalase-negative rods were further identified using API 50CHL (bio-Merieux, Marcy l’Etoile, France) and MALDI-TOF MS analysis (Bruker, Billerica, Massachusetts, USA). Of 26 isolates, 4 strains of *Lactobacillus plantarum* and 4 strains of *Lactobacillus brevis* were selected for further study. One of the selection criteria was the growth capacity of these strains in the presence of bile salts and gastric juice which was checked by methods described by Li et al. [22].

*P. mirabilis* (strain C7) was derived from encrusted biofilm formed on urinary catheters of long-term catheterized patients and was deposited in the bacterial strain collection at the Department of Biology of Bacteria, the University of Lodz. The method of isolation of this strain and its characteristics had been described in our previous study [31].

### Adhesion Assay

The ability of the *Lactobacillus* strains to adhere to both intestinal (Caco-2) and urinary (Hu609) epithelial cells was determined. In this assay, the cells were grown in a suitable medium line, in the case of Caco-2, it was Dulbecco’s medium; for Hu 609, RPMI 1640 medium was used. Media were supplemented with 10% heat-inactivated fetal calf serum (FCS, Lonza, Walkersville, MS, USA), 2 mM ultraglutamine (Lonza), 100 IU/mL penicillin, and 100 μg/mL streptomycin (Polfa Tarchomin, Warsaw, Poland). For the adhesion assay, all types of epithelial cells (1 × 10^5^ cells per well) were seeded into 24-well plates and grown for 24 h in a humidified incubator with 5% CO_2_ at 37 °C in culture medium supplemented only with 10% fetal bovine serum (FCS, Lonza) and 2 mM ultraglutamine (Lonza). Afterwards, the cells were infected with 1-mL bacterial suspension (10^8^ CFU/mL in culture medium with 10% FBS + 2 mM ultraglutamine) (Lonza). After 3-h incubation (37 °C, 5% CO_2_), the suspension was aspirated, and the cells were washed three times with PBS (pH 7.3) to remove free bacteria. To establish the number of adherent bacteria, a monolayer was lysed using 1% Triton X-100. After that, 1/10 dilution of the suspension was seeded on MRS plates and incubated for 24 h at 37 °C. The results are expressed as bacterial colony-forming units (CFU) recovered per milliliter.

### Antibacterial Activity

The antimicrobial activity of *Lactobacillus* strains was checked by broth microdilution method. Cultures of *Lactobacillus* (MRS broth for 48 h at 37 °C) were centrifuged 8000×*g* for 20 min at 4 °C. The supernatant without the bacteria was recovered and separated into two aliquots. One of them was untreated; the second supernatant was neutralized to pH 6.5 with 1 N sodium hydroxide (NaOH). In the case of untreated supernatants, the pH ranged between 3.42 and 3.86. The supernatants were sterilized by filtration through Minisart® (Sartorius, Goettingen, Germany) syringe filters 0.2 μm. The antibacterial activity of *Lactobacillus* was checked against *P. mirabilis* C7. The strain was cultured in TSB medium (tryptic soy broth, BTL, Warsaw, Poland) for 24 h at 37 °C. In the experiment, the dilutions of supernatants were prepared in F-bottom 96-well plates. To each well, 100 μl of bacterial suspension (10^5^ CFU/mL) in TSB to 100-μl tested supernatant was added. As controls, 200 μl TSB (negative control) and 200 μl bacterial suspension in TSB of the tested bacterial strains (positive control) were used. Inhibition of bacterial growth was detected by measuring turbidity at 600 nm using a microplate reader Multiskan Ex (Labsystems, Helsinki, Finland).

### Analysis of the Influence of *Lactobacillus* on the Crystallization Caused by *P. mirabilis*

The effect of *Lactobacillus* on crystallization caused by *P. mirabilis* was analyzed in eight independent tests. The first probe (control) contained only *P. mirabilis*, in the remaining seven tests, *P. mirabilis* bacteria were incubated simultaneously with individually tested strains of *Lactobacillus*. *Lactobacillus* strains were cultured on MRS medium (De Man, Rogosa and Sharpe broth, BTL, Warsaw, Poland) for 48 h at 37 °C, while *P. mirabilis* was grown in a TSB medium (tryptic soy broth, Warsaw, BTL, Poland) for 18 h at 37 °C. Before each experiment, a suspension of both or only one species in 20 mL of synthetic urine was prepared. The number of bacteria per milliliter was determined spectrophotometrically at 600 nm (Ultrospec 2000, Pharmacia Biotech, Vienna, Austria), where the density of the suspension was adjusted to 1–5 × 10^5^ bacteria per milliliter. Synthetic urine is a composition which corresponds to the mean concentration of the mineral components found during a 24-h period in normal human urine and was used in in vitro studies of uropathogens [19, 44]. The solution was prepared using the modified method previously described by McLean et al. [30] and consisted of the following components (g/L): urea (CH_4_N_2_O)—25.0, sodium chloride (NaCl)—4.6, potassium dihydrogen phosphate (KH_2_PO_4_)—2.8, sodium sulfate (Na_2_SO_4_)—2.3, potassium chloride (KCl)—1.6, ammonium chloride (NH_4_Cl)—1.0, creatine (C_4_H_9_N_3_O_2_)—1.1, calcium chloride dihydrate (CaCl_2_ × 2H_2_O)—0.651, magnesium chloride hexahydrate (MgCl_2_ × 6H_2_O)—0.651, sodium citrate (Na_3_C_6_H_5_O_7_)—0.65, sodium oxalate (Na_2_C_2_O_4_)—0.02 (Sigma, Poznan, Poland), and tryptic soy broth, 10.0 (BTL, Warsaw, Poland). After adjusting the pH to 5.8, the synthetic urine solution was sterilized by passing through a 0.2-μm pore-size filter (Minisart® Sartorius, Goettingen, Germany).

Bacterial suspension in synthetic urine was incubated without mixing for 24 h at 37 °C. At the beginning of the experiment (0 h) and after 5, 8, and 24 h, incubation pH, bacterial viability, and the degree of crystallization were evaluated in all cultures. A pure culture of *P. mirabilis* was used as a control system in all mentioned test. The numbers of bacteria in pure and mixed cultures, defined as CFU/mL (colony forming units per milliliter), were determined on McConkey medium (BTL, Warsaw, Poland) and MRS agar for *P. mirabilis* and *Lactobacillus*, respectively.

Crystallization was determined by spectrophotometric measurements, microscopic observation using direct phase-contrast microscopy (Nikon Eclipse 2000S, Tokyo, Japan), and its intensity was tested by chemical analysis. The turbidity of bacterial suspension in synthetic urine as an absorbance of light at 600 nm wavelength was measured (Ultrospec 2000, Pharmacia Biotech, Uppsala, Sweden). For chemical analyses, a sample (1 mL) of crystals with a bacterial suspension was centrifuged at 8000×*g* for 10 min. The obtained pellet was suspended in aqueous solution of nitric acid (30% HNO_3_, POCh, Gliwice, Poland) and incubated for 60 min at 100 °C. After mineralization, calcium and magnesium concentrations were determined by atomic absorption spectroscopy (AAS, SpectrAA-300 Varian, Palo Alto, USA). X-ray powder diffraction (XRD) was used for phase identification of a crystalline material. Analysis was performed on X’pert PRO MPD (PANalytical) diffractometer where CuKα radiation monochromatized by nickel filter was applied. Measurements were done in the range of 2θ angles from 3 to 90° with a continuous scan (step 0.0167°) where the measurement time of one step was 30 s. The X’Pert High Score Plus PANanalytical (Almelo, The Netherlands) software was used for indexing peaks in the XRD pattern.

For the evaluation of bacterial extracellular protein and polysaccharide content, 1 mL of each sample was taken at 0, 5, 8, and 24 h of incubation and centrifuged at 8000×*g* for 10 min in room temperature. Protein concentrations were determined by the method of Lowry et al. [24] using bovine serum albumin as a standard. The phenol-sulfuric acid method described by Masuko et al. [27] with glucose as a standard was selected for carbohydrate determination.

### Statistics

The data are presented as mean ± standard deviation (SD) of three to five independent experiments. Statistical analyses were based on the Mann-Whitney *U* test performed using Statistica software version 13.3 pl (StatSoft, Krakow, Poland). The results were considered to be statistically significant at *p* < 0.05. Statistical differences between groups are indicated in the text.

## Results

### *Lactobacillus* strains Characteristics

Eight strains selected for this study were characterized in terms of their antibacterial activity and ability to colonize the epithelium both of the urinary tract and the digestive system. As shown in Table 1, the tested strains showed the ability to adhere both to the Caco-2 and Hu 609 lines. The number of bacteria adhering to the urinary epithelium was varied and ranged from 14.3 to 136.85 × 10^5^ CFU/mL. Similarly, in the case of the Caco-2 line, the number of adherent bacteria was 19.0 to 330.4 × 10^5^ CFU/mL. Only S18K7 and S21K7 strains strongly adhered to both Caco-2 and Hu 609 lines. As for the antibacterial activity, untreated supernatants of all *Lactobacillus* strains inhibited the growth of *P. mirabilis* C7. After the neutralization of the pH of 1 N NaOH, the inhibitory activity remained in the supernatants of the *L. brevis* strains S1K1, S20K3, and S21K5 and only in one strain of *L. plantarum* S23K2, which indicated the presence of antibacterial agents other than organic acids.

**Table 1:** Characteristic of chosen *Lactobaccilus* strains isolated from fermented cucumbers

Species	Strain	Antibacterial activity of supernatants^a^		Ability of adhesion^b^ (CFU/mL × 10^5^)	
		untreated	treated 1N NaOH	Caco-2	Hu609
*L. brevis*					
	S1K1	+	+	25.1 ± 7.2	14.3 ± 3.9
	S18K7	+	-	100.1 ± 30.0	107.4 ± 62.1
	S20K3	+	+	27.1 ± 11.7	45.6 ± 14.3
	S21K5	+	+	19.0 ± 6.5	25.1 ± 11.9
*L. plantarum*					
	S2K18	+	-	53.2 ± 10.8	44.8 ± 10.2
	S18K2	+	-	330.4 ± 131.7	79.3 ± 17.1
	S21K7	+	-	101.1 ± 39.6	136.9 ± 58.4
	S23K2	+	+	39.4 ± 18.1	23.9 ± 9.5

^a^- antibacterial effect determined by microdilution method in relation to *P. mirabilis* C7, a 99% decrease in absorbance compared to the positive control was considered as a positive result; ^b^adhesion expressed as the number of bacteria adhering to the epithelium after 3 hours of incubation

### Effect of *Lactobacillus* on Bacterial Growth and Crystallization Caused by *P. mirabilis* in Synthetic Urine

#### Growth of Bacteria

**Fig. 1:**
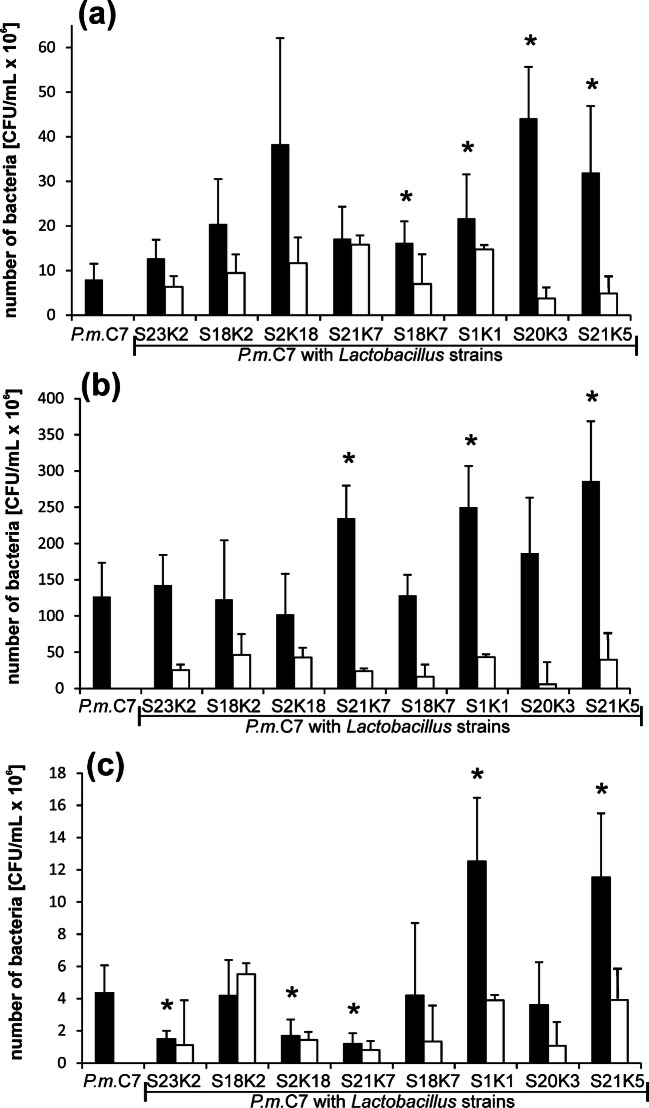
Growth of *P mirabilis* and *Lactobacillus* spp. in mixed cultures during incubation in synthetic urine after 5 h (**a**), 8 h (**b**), and 24 h (**c**). The values represent means ± SD of 5 experiments. *p ≤ 0.05 comparing *P. mirabilis* cells number in mixed cultures with those in pure culture; black bars—number of *P mirabilis* cells, white bars—*Lactobacillus* cells number

In the crystallization experiment, *Lactobacillus* strains were simultaneously incubated with the *P. mirabilis* strain in synthetic urine. Changes in *P. mirabilis* growth intensity during incubation are shown in Fig. 1. The initial suspension for *P. mirabilis* was 1–1.4 × 10^6^ CFU/mL, and for *Lactobacillus* spp*.,* it was between 0.9 and 1.6 × 10^6^ CFU/mL depending on the experiment. With the time of incubation, the number of *P. mirabilis* cells increased reaching in 5 h in mixed cultures with *Lactobacillus*, the values were from 12.6 × 10^6^ CFU/mL (*L. plantarum* S23K2) to 38 × 10^6^ CFU/mL for the co-culture with *L. plantarum* S2K18. At that point of the experiment, *P. mirabilis* growth was higher in the presence of lactobacilli compared with the growth in the pure culture, but this difference was not statistically significant in each case (Fig. 1). Up to 8 h of incubation, the most intensive growth of *P. mirabilis* was observed reaching the values in the range of 102– 266 × 10^6^ CFU/mL both in the mixed cultures and in the pure culture. The number of *P. mirabilis* cells did not differ between the mixed and pure cultures as it was seen in 5 h, except for the co-culture with *L. plantarum* S21K7 and *L. brevis* S1K1 and S21K5, where *P. mirabilis* grew more intensively. At that point, the culture pH was already very high (Table 2), and the viability of the bacteria started to decrease. After 24 h of incubation, the number of *Proteus* bacilli in the culture varied considerably depending on the *Lactobacillus* strain with which it was grown. In the case of the co-culture with *L. plantarum* S23K2 and S21K7, the number of *P. mirabilis* cells was lower and reached 1.48 × 10^6^ and 1.2 × 10^6^ CFU/mL, respectively, in comparison with the pure *P. mirabilis* culture (4.36 × 10^6^ CFU/mL). On the other hand, *L. brevis* S1K1 and S21K5 promoted the growth of *P. mirabilis* in the same conditions because the number of *P. mirabilis* bacteria reached 12.5 × 10^6^ and 11.5 × 10^6^ CFU/mL, respectively. All *Lactobacillus* strains tested in mixed cultures with *P. mirabilis* survived until the end of the experiment, and the kinetics of growth of the lactobacilli was similar to that of *P. mirabilis*. The number of bacteria increased until 8 h of incubation, when it reached the highest value (6.04–46.33 × 10^6^ CFU/mL), and then it decreased, and in 24 h, there were about 10^5^. The S18K2 strain exhibited the most intensive growth, while the S20K3 strain probably survived, but its cells did not divide in this environment.

**Table 2:** pH changes in *P. mirabilis* culture with or without *Lactobacillus* strains in synthetic urine during incubation at 37 °C

Culture	pH value			
	0 h	5 h	8 h	24 h
*P. mirabilis* C7	6.18 ± 0.36	6.65 ± 0.35	7.5 ± 0.64	10.01 ± 0.08
*P. mirabilis* C7 + *L. plantarum* S23K2	6.20 ± 0.36	6.69 ± 0.38	7.82 ± 0.82	9.99 ± 0.10
*P. mirabilis* C7 + *L. plantarum* S18K2	6.18 ± 0.34	6.60 ± 0.38	7.45 ± 0.89	9.84 ± 0.34
*P. mirabilis* C7 + *L. plantarum* S2K18	6.50 ± 0.06	6.94 ± 0.05	8.27 ± 0.39*	10.03 ± 0.01
*P. mirabilis* C7 + *L. plantarum* S21K7	6.51 ± 0.01	6.92 ± 0.07	8.25 ± 0.34*	10.01 ± 0.01
*P. mirabilis* C7 + *L. brevis* S18K7	6.53 ± 0.02	6.89 ± 0 02	8.24 ± 0.34*	10.02 ± 0.01
*P. mirabilis* C7 + *L. brevis* S20K3	6.18 ± 0.10	6.60 ± 0.13	8.20 ± 0.27*	9.68 ± 0.06*
*P. mirabilis* C7 + *L. brevis* S21K5	6.16 ± 0.06	6.62 ± 0.14	8.26 ± 0.24*	9.68 ± 0.05*
*P. mirabilis* C7 + *L. brevis* S1K1	6.17 ± 0.09	6.61 ± 0.13	8.19 ± 0.26*	9.66 ± 0.06*

Results are presented as means ± standard deviation (SD) of five experiments

*p < 0.05 for comparison of pH in mixed culture vs. pure culture of *P. mirabilis*, Mann-Whitney *U* test

#### Changes in pH During the Co-Culture of *P. mirabilis* with *Lactobacillus* Spp*.*

During the growth of *P. mirabilis* in synthetic urine, the pH increases due to the hydrolysis of urea. In this experiment, the pH changes in the pure culture of *P. mirabilis* were compared to those observed in the mixed cultures (*P. mirabilis* + *Lactobacillus sp.*). The starting urine pH ranged from 6.16 to 6.53 and increasing all the time; in 24 h, it reached a value close to 10 (Table 2). After 5 h, an increase in pH was already observed; the highest value to about 7 was noted for samples with the mixed cultures of *P. mirabilis* from *L. plantarum* S2K18 and S21K7 and *L. brevis* S18K7. After 8 h of incubation, the differences between the samples were more visible. In most cases, the pH in the mixed cultures was higher than that in the pure cultures. The exception was the cultures with *L. plantarum* S23K2 and *L. plantarum* S18K2 where the pH value was close to that of the pH of the pure culture. At the end of the experiment, significantly lower pH values compared with the *P. mirabilis* culture were noted in co-cultures with 3 *L. brevis* strains S20K3, S21K5, and S1K1.

#### Effect of *Lactobacillus* Spp. on the Crystallization Caused by *Proteus mirabilis*

**Fig. 2:**
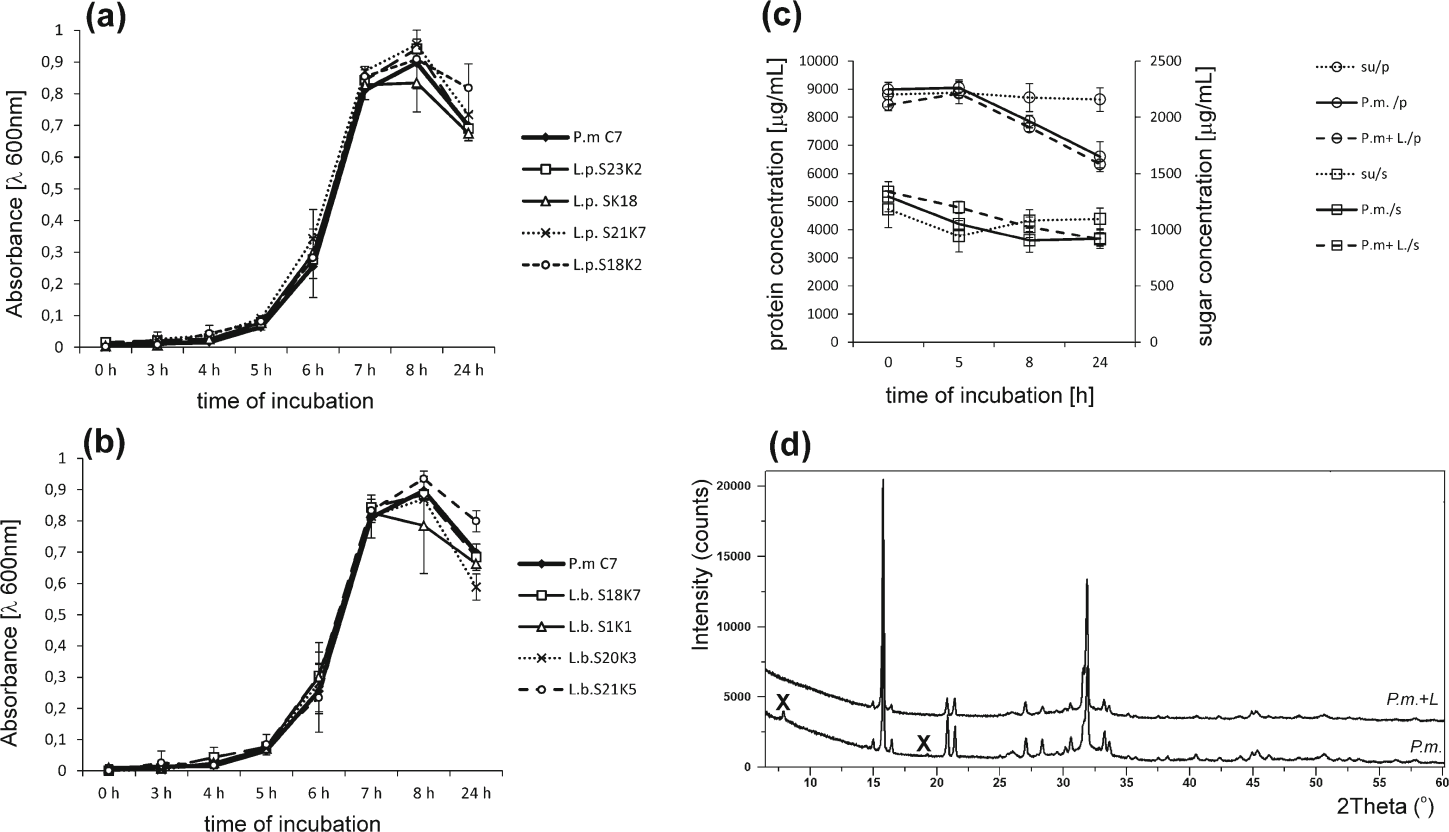
Crystallization intensity in *P. mirabilis* culture and mixture of *P.* mirabilis with *L. plantarum* (**a**), and *L. brevis* (**b**) evaluated spectrophotometrically; changes in concentration of sugar and proteins in supernatants of pure and mixed cultures during incubation time (values for mixed cultures are the average of all samples from *Lactobacillus* spp.); su synthetic urine, P.m. *P mirabilis* culture, P.m. + L- (**c**) and comparison of X-ray diffraction pattern of the sample from *P. mirabilis* culture with the samples from culture of *P. mirabilis* + *L. plantarum* S23K2 (difference samples are marked X) (**d**)

**Fig. 3:**
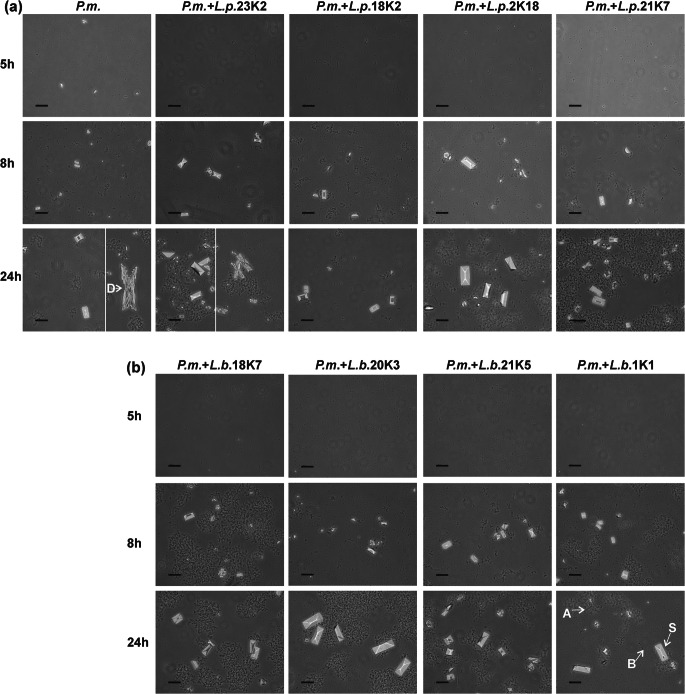
Crystallization caused by *P. mirabilis* during incubation for 5 h and 24 h in synthetic urine in the co-culture with *Lactobacillus* *plantarum *(**a**) and (**b**) *Lactobacillus brevis* strains; B bacterial cells, A apatite, S struvite, D dendrites of struvite. Bar scale 40 μm

Along with the increase in pH resulting from the decomposition of urea, calcium and magnesium phosphates fall out of the urine solution and, at a later stage, crystallize as apatite and struvite. Apatites (calcium phosphates) appear when the pH exceeds 6.8 while struvite (ammonium magnesium phosphate) when the pH exceeds 7.5. Crystallization can be evaluated spectrophotometrically because it is accompanied by an increase in turbidity of suspension. As shown in Fig. 2a, b, the time course of the curves is very similar for all samples tested including the control and mixed cultures of *P. mirabilis* with *L. plantarum* or *L. brevis*. The increase occurred from 5 h reaching the maximum value in 8 h and then the absorbance slowly decreased. In 8- and 24-h incubations, differences in the intensity of crystallization between mixed cultures and control were visible. In the case of the culture containing *L. brevis*, the absorption for 3 strains was below this value for the pure culture of *P. mirabilis*. For the *L. plantarum* cultures, this tendency was opposite; the absorbance for most suspensions was equal to or higher than the absorbance of the pure culture. The samples were submitted to a more detailed analysis at 0, 5, 8, and 24 h. Microscopic observation showed that the first small crystals appeared at about 5 h of the experiment (Fig. 3). After 8 h of incubation, amorphous apatite precipitates and struvite crystals with typical hemimorphic morphology of coffin-lid shape along with bacterial cells were observed in all samples. Crystallization occurred all the time, and after 24 h, an increase in the number and size of single crystals was noted. For the *P. mirabilis* monoculture and the mixed culture of *P. mirabilis* and *L. plantarum* S23K2, dendrite crystals, demonstrating faster growth, were also visible. It is interesting that in most samples from the mixed cultures both in 5 h and 24 h, the crystals were larger in size compared to the pure *P. mirabilis* culture, which was especially visible when *L. plantarum* S2K18 and *L. brevis* S20K3 and S1K1 were present in the culture. An attempt was made to determine the presence of proteins and polysaccharides in post-culture supernatants by identifying the factor responsible for the formation of larger crystals. However, the concentrations of polysaccharides and proteins were comparable in all tests (Fig. 2c). The concentration of polysaccharides was 1315.6 μg/mL at Oh, and from 5 h, it began to decline to average values of 1050.3 μg/mL and 1198.4 μg/mL for *P. mirabilis* culture and mixed cultures. respectively, reaching the lowest level at 24 h, where the decrease was about 30% (921.5 μg/mL and 915.3 μg/mL for “*P. mirabilis*” and “*P. mirabilis* + *Lactobacillus*”, respectively) compared with the initial value in the cultures. Similarly, the protein concentration was the highest at 0 h (8703.3 μg/mL); from 8 h, it began to decline till the end of the experiment (6596.2 μg/mL (*P. mirabilis*) and 6325.3 μg/mL (*P. mirabilis* + *Lactobacillus*). The decrease in the content of polysaccharides and proteins in the supernatants compared with the sterile medium results from the use of these components by bacteria as a source of carbon and energy. This indicated a similar metabolic activity of bacteria in all cultures tested rather than the secretion of substances that could modulate the crystallization process. The intensity of crystallization was also quantified by analyzing the calcium and magnesium levels in the samples. As shown in Table 3, the levels of these cations did not differ significantly between 0- and 5-h incubation, which indicated a very low level of salt crystallization and confirmed the results of the previous analyses at 5 h. Calcium and magnesium levels significantly (on average 10 times) increased after 8 h of incubation. The content of these ions in the formed crystals was higher in the mixed cultures than in the pure culture, and only in the case of the *P. mirabilis* + *L. plantarum* S23K2 sample, it was comparable with the culture containing *P. mirabilis* alone. The intensity of crystallization increased with the incubation time, but not as intensely as between 5 and 8 h of incubation, reaching values between 41.12 and 56.11 μg/mL for calcium and 35.42 and 53.08 μg/mL for magnesium at the end of the experiment. To confirm the composition of the crystalline components in the samples, XRD analysis was performed after 24-h incubation. As shown in Fig. 2d, in 2 samples with *P. mirabilis* and *P. mirabilis* with *L. plantarum* S23K2 (a representative sample for the mixed cultures), the obtained samples had a crystalline character and the diffraction pattern was similar. The dominant components such as struvite and hydroxyapatite were also found in both types of culture. The only significant difference in the phase composition of both samples was the presence of a small amount of hydrated calcium phosphate in the sample containing *P. mirabilis* alone*.*

**Table 3:** Crystallization intensity occurring in synthetic urine in the presence of *P. mirabilis* or *P. mirabilis* with various *Lactobacillus* strains during incubation at 37 °C for 24 h

Culture	Amount of calcium and magnesium (μg/mL)							
	0 h		5 h		8 h		24 h	
	Ca^2+^	Mg^2+^	Ca^2+^	Mg^2+^	Ca^2+^	Mg^2+^	Ca^2+^	Mg^2+^
*P.m*	2.28 ± 0.47	1.11 ± 0.19	2.97 ± 0.33	1.22 ± 0.24	23.02 ± 4.39	17.26 ± 7.09	41.12 ± 6.75	47.68 ± 8.9
*P.m* + *L.p*. 23K2	2.66 ± 0.34	1.24 ± 0.22	2.97 ± 0.43	1.31 ± 0.36	21.37 ± 5.05	18.24 ± 9.7	53.71 ± 6.67*	45.66 ± 10.4
*P.m.* + *L.p*. 18K2	3.05 ± 0.39	1.21 ± 0.27	3.21 ± 0.37	1.2 ± 0.36	37.9 ± 5.15*	20.19 ± 9.22	56.11 ± 7.0*	35.42 ± 13.4
*P.m*. + *L.p.* 2K18	2.75 ± 0.88	1.22 ± 0.35	2.86 ± 0.47	1.11 ± 0.2	40.68 ± 7.74*	33.55 ± 2.17*	49.7 ± 7.74	48.13 ± 8.78
*P.m*. + *L.p* 21K7	2.1 ± 0.3	0.92 ± 0.2	2.73 ± 0.57	1.13 ± 0.29	42.03 ± 4.87*	43.08 ± 10.19*	49.91 ± 5.62	46.02 ± 7.8
*P.m*. + *L.b.* 18K7	2.28 ± 0.3	1.06 ± 0.1	3.36 ± 0.83	1.2 ± 0.59	42.68 ± 6.15*	31.46 ± 17.81	45.19 ± 10.35	53.05 ± 8.16
*P.m*. + *L.b*. 20K3	4.39 ± 1.01	1.54 ± 0.6	3.99 ± 1.83	1.1 ± 0.23	33.34 ± 5.4*	39.8 ± 9.64*	41.73 ± 3.05	47.79 ± 8.27
*P.m*. + *L.b*.21K5	4.45 ± 1.84	1.3 ± 0.4	4.57 ± 1.57*	0.79 ± 0.37	33.73 ± 2.77*	44.07 ± 8.4*	41.13 ± 2.75	53.08 ± 8.45
*P.m*. + *L.b*. 1K1	3.64 ± 0.78	1.48 ± 0.54	4.75 ± 1.4*	1.26 ± 0.3	35.26 ± 2.91*	41.43 ± 5.19*	39.28 ± 2.64	48.36 ± 12.18

*P.m. Proteus mirabilis C7*, *L.p. Lactobacillus plantarum*, *L.b. Lactobacillus brevis*

*p < 0.05 for comparison of each ion concentration to control culture of *P. mirabilis*, Mann-Whitney *U* test

## Discussion

In recent years, interest in the use of probiotic strains for the treatment or prevention of urinary tract infections has increased. To date, the results are not clear as to the effectiveness of the antibacterial effect of probiotics, and this is probably the result of the selection of concepts and the route of administration to patients. Strains used for treatment are usually isolated from human stool, or in the case of women, they come from the genitourinary tract [17, 41]. However, food-derived strains with high antibacterial properties might also play an important role. Manzoor et al. [26] showed antimicrobial activity of *Lactobacillus* spp. strains including *L. plantarum* and *L. brevis* isolated from local food against antibiotic resistant uropathogens including *E. coli*, *K. pneumoniae*, and *E. faecalis*. Similarly, Liu et al. [23] evaluated the effect of lactic acid bacteria isolated from fermented food on uropathogenic *E. coli* and showed that in both in vitro and in vivo conditions, the ability of the pathogen to adhere and its growth in the presence of LAB decreased. *Lactobacillus* spp. are also the main component of urobiome, i.e., the microbiome of the urinary tract, where they play an important role in maintaining the homeostasis of this environment [4, 21]. Their elimination from natural flora results in greater susceptibility to infection and the development of other diseases of the genitourinary system. The aim of this study was examining the effect of two *Lactobacillus* species, *Lactobacillus plantarum*, and *Lactobacillus brevis*, derived from fermented foods on crystallization induced by *P. mirabilis* during urinary tract infection. Strains with different antimicrobial activity were selected for the study. We assumed that those exhibiting antimicrobial properties would either act in a bactericidal manner on the *P. mirabilis* or inhibit the activity of its virulence factors, thus affecting crystallization. However, none of them inhibited the growth of *P. mirabilis* in synthetic urine or crystallization. This may be due to the inhibition of the ability to synthesize antibacterial compounds by *Lactobacillus* spp. in synthetic urine or to the reduced activity of *Lactobacillus*-produced organic acids caused by the high urine pH. Based on their experience, Yang et al. [46] showed that the ability to produce bacteriocins depends on the pH, temperature, and composition of the substrate. In addition, Mataragas et al. [28] stated that the highest bacteriocin production occurs not under optimal growth conditions but with reduced temperature and pH. In our experiments, *Lactobacillus* not only did not inhibit growth but even caused a significantly larger growth in *P. mirabilis* compared with the control. This is not the first case where co-cultivation promotes the growth of one or all microorganisms in the co-culture. Alteri et al. [3] studied in vivo co-infections *E. coli* and *P. mirabilis* on a mouse model. Based on their research results, they concluded that in mixed infection, both *E. coli* and *P. mirabilis* colonize the urinary tract of mice better than in mono infection, which results from the metabolic properties of these microorganisms consisting in the complementary use of carbon sources. Similar results were obtained in our earlier studies where a better growth of *P. mirabilis* in vitro in synthetic urine was observed in the presence of other uropathogens including *E. coli* [43]. Therefore, it cannot be ruled out that in this case, *P. mirabilis* uses other sources of carbon and energy than *Lactobacillus*, or *Lactobacillus* provides nutrients for *P. mirabilis*. Even more interesting is the growth of *Lactobacillus* in our experimental condition, although *L. plantarum* and *L. brevis* grow in human urine, and they are isolated from the urinary tract; in this case, during the growth of *P. mirabilis*, the pH of synthetic urine increases significantly (up to 10), which is not typical for this group of bacteria. Sawatari and Yokota [37] studied the effect of alkaline stress on the growth, physiology, and metabolism of lactobacilli isolated from various sources including human feces, saliva, or food. Maximum pH allowing the growth of these lactobacilli ranged between 6.7 and 8.9, with pH values of 8.5 for strains derived from plants (*L. casei*, *L. paracasei* subsp. *tolerans*, *L. paracasei* subsp. *paracasei*, *L. curvatus*, *L. pentosus*, and *L. plantarum*). They found that lactobacilli growth in an alkaline environment depended on the alkali tolerance of the cellular metabolism, especially on the glycolysis reactions. It is possible that these mechanisms of survival in the alkaline environment also occurred in the strains used for the present study.

The aim of this work was to find a *Lactobacillus* strain whose properties would allow inhibiting the growth of *P. mirabilis* and/or crystallization of mineral salts of urine induced by these bacteria. However, as previously mentioned, *Lactobacillus* enhanced the growth of *P. mirabilis*, and some of the tested strains increased crystallization. The increase in the intensity of crystallization was manifested by both the formation of bigger crystals and the crystallization of larger amounts of calcium and magnesium phosphates. The intensity of crystallization in such conditions may depend on the rate of pH increase, which affects the degree of supersaturation of urine [9]. The increase in the concentration of ammonium ions and pH play a key role in inducing struvite and apatite crystallization, and in the presence of the appropriate concentration of calcium and magnesium, ions also have an impact on the crystallization rate [40]. This is confirmed by our results; in mixed cultures of *P. mirabilis* with *Lactobacillus* (except for cultures with *Lactobacillus* S23K2 and S18K2) at the 8 h of incubation, both the pH and the amount of crystallized calcium and magnesium salts were higher than in with *P. mirabilis* monoculture. The intensity of crystallization also depends on the presence of bacterial factors, such as extracellular polysaccharides or other macromolecules. Bacterial macromolecules having different binding capacity for calcium and magnesium cations can accelerate or inhibit crystallization [13]. These compounds possessing a strong affinity bind cations from the environment and act as crystallization inhibitors; on the other hand, ones with weak affinity easily release cations promoting their accumulation and subsequent crystallization [13, 45]. Like other bacteria, due to the presence of cell wall components, e.g., teichoic acids, proteins, and polysaccharides, lactic acid bacteria may have a surface charge that allows interaction with ions contained in the environment. In addition, exopolysaccharides as hetero- and homopolysaccharides are produced in the environment in very large quantities by many *Lactobacillus* strains including *L. brevis* and *L. plantarum* [12]. The ability to bind calcium ions by *Lactobacillus* exopolysaccharides has been experimentally confirmed by Astasov-Frauenhoffer et al. [6] in studies on the role of exopolysaccharides of *Lactobacillus plantarum* and *Lactobacillus paracasei* in binding calcium in cariogenic biofilms. Taking this into account, it was verified whether there is a difference in the level of macromolecules such as proteins and polysaccharides in the culture medium. Unfortunately, no differences in total amount of polysaccharides and proteins between samples from *P. mirabilis* monocultures and co-culture with different *Lactobacillus* strains were shown. The participation of extracellular macromolecules as well as those associated with the surface of lactobacilli in crystallization cannot be excluded, but it requires more detailed analysis, especially since there is evidence that the proteins of this microbial group participate in crystallization. Borah et al. [11] have shown that the acidic proteins of *L. plantarum*, *L. acidophilus*, and *Pediococcus acidilactici* affect crystallization by providing nucleation centers and changing the morphology of calcium and barium salt crystals.

In summary, it has been shown that *P. mirabilis*, co-cultured with *L. plantarum* and *L. brevis* strains derived from food and with potential to colonize the urinary tract, shows better viability and resistance to high pH. Such conditions also proved to be conducive to crystallization of calcium and magnesium salts induced by *P. mirabilis*. These interactions between microorganisms along with an explanation of the effect on the intensity of crystallization require further investigation; however, at this stage, it indicates that without knowing exactly the complexity of the interaction of microorganisms from different environments, it is not safe to introduce bacteria into the human body as a means of preventing or treating diseases.
